# Molecular Evolution and Pathogenicity of Methicillin-Resistant *Staphylococcus aureus*

**DOI:** 10.3390/antibiotics13100953

**Published:** 2024-10-10

**Authors:** Kunyan Zhang

**Affiliations:** 1 Department of Pathology & Laboratory Medicine, University of Calgary, Calgary, AB T2N 1N4, Canada; 2 Department of Microbiology, Immunology & Infectious Diseases, University of Calgary, Calgary, AB T2N 1N4, Canada; kzhang@ucalgary.ca; 3 Department of Medicine, University of Calgary, Calgary, AB T2N 1N4, Canada; 4 Centre for Antimicrobial Resistance, Alberta Health Services/Alberta Precision Laboratories/University of Calgary, Calgary, AB T2N 1N4, Canada; 5 The Calvin, Phoebe and Joan Snyder Institute for Chronic Diseases, University of Calgary, Calgary, AB T2N 1N4, Canada

*Staphylococcus aureus* is a Gram-positive and coagulase-positive pathogen, belonging to the *Staphylococcaceae* family. It has the capability to acquire resistance to most antibiotics and to collect virulence factors [1,2,3]. This ability is further augmented by the constant emergence of new clones [1,4]. Historically, penicillin-resistant *S. aureus* emerged in 1942 within two years of the introduction of penicillin [5,6,7,8]. A semi-synthetic antibiotic, methicillin, was then developed to act as a substitute for the treatment of penicillin-resistant *S. aureus*. However, methicillin-resistant *S. aureus* (MRSA) was clinically identified in 1960 shortly after its introduction in 1959 [9]. Thereafter, worldwide outbreaks of MRSA have occurred in waves [10,11,12,13,14,15,16,17,18,19,20,21,22,23,24]. The dissemination of MRSA is marked by the propagation of a number of clones harboring specific genetic backgrounds in different continents [1,18,25,26,27,28,29,30,31,32,33]. Although most MRSA strains are hospital-acquired originally, community-associated strains (CA-MRSA) have now been increasingly recognized worldwide and are both phenotypically and genotypically different from hospital-associated (HA)-MRSA [1,34,35,36,37,38,39,40]. The importance of livestock-associated (LA)-MRSA has also been frequently reported since the mid-2000s [41,42,43]. Infections due to MRSA, in particular CA-MRSA and LA-MRSA, are associated with more severity and higher mortality rate compared to infections caused by methicillin-susceptible strains [22,44,45,46,47,48].

Staphylococci consist of more than 45 staphylococcal species (*Staphylococcus* spp.), especially coagulase-negative staphylococci (CoNS). Although most CoNS are harmless and exist as opportunistic pathogens on the skin and mucous membranes of human and other animals, their significance has been boosted with an increasing number of CoNS infections identified, in particular their role in the evolution and pathogenicity of MRSA [49,50,51,52].

In this Special Issue, there were a total of 13 papers including 10 research articles [Contributions 1,3,5–8,10–13] and 3 review/perspective articles [Contributions 2,4,9], with a wide spectrum of staphylococcal research, covering the latest advances in molecular epidemiology, evolution, and pathogenicity of staphylococci.

MRSA molecular epidemiological data from less developed countries are limited. In this Special Issue, Ullah et al. [Contribution 1] described an emerging MRSA strain, ST113-MRSA-IV, which is closely related to ST8 and multi-drug resistance in Pakistan and provided detailed genomic comparative information for this strain. Chai et al. [Contribution 3] investigated the prevalence, antibiogram, and genomic characteristics of methicillin-susceptible *S. aureus* (MSSA) and MRSA isolated from animal handlers in Peninsular Malaysia and provided background information for further studies on the transmission of *S. aureus* between animals and humans. Hwang [Contribution 11] showed a general distribution of the major MRSA strains in the Republic of Korea from 1994 to 2020.

For LA-MRSA, LeÃ£o et al. [Contribution 6] reported the emergence of a *cfr*-mediated linezolid-resistant LA-MRSA strain, ST398-t011-MRSA-Vc, from healthy pigs in Portugal. Iurescia et al. [Contribution 8] investigated the genomic variants in association with the linezolid-resistant phenotype in the *cfr*-mediated linezolid-resistant LA-MRSA isolates from Italian pig farms. These studies implied a transmission risk from livestock to humans by the presence of *cfr*-positive LA-MRSA and indicated the importance of continuous genomic surveillance of *cfr*-positive LA-MRSA.

Plasmids and phagemids play a crucial role in MRSA evolution and adaptation, as well as the acquisition and spread of antimicrobial resistance and virulence genes. Al-Trad et al. [Contribution 10] explored the plasmid profiles of the clinical MRSA isolates during the period from 2016 to 2020 obtained from a tertiary hospital in the state of Terengganu, Malaysia. Saei et al. [Contribution 12] gave details of the role of prophage ϕSa3 in the adaption of *S. aureus* ST398 sub-lineages from human to animal hosts.

In the virulence realm, Pulia et al. [Contribution 5] studied the staphylococcal virulence gene’s expression in situ in human skin and soft tissue infection patients from two medical centers in Wisconsin, USA, and demonstrated a relative increase in the transcripts of several toxins, adhesion, and regulatory genes. Kim et al. [Contribution 7] used DNA affinity capture assay (DACA) to study the MRSA virulence factor and antibiotic resistance regulation. They showed that the SarA protein bound to all *mecA*, *sarA,* and *sarR* promoters, and the *sarA* truncated mutant weakened antibiotic resistance to oxacillin and reduced biofilm formation. Phenol-soluble modulin (PSM) belongs to the peptide toxins superfamily and possesses similar alpha-helical and amphipathic secondary structures. It plays significant roles in the pathogenesis of *S. aureus* and *S. epidermidis* through its pro-inflammatory, cytolytic, and biofilm-structuring functions. The methicillin resistance-associated PSM locus (*psm*-*mec*) is found in the class A *mec* gene complex within the staphylococcal chromosome cassette *mec* (SCC*mec*) in many staphylococcal species. Cheung et al. [Contribution 13] characterized the SCC*mec* elements from methicillin-resistant *S. pseudintermedius* (MRSP) isolates representing the four major lineages in the United States and gained insights into the composition of SCC*mec* elements in MRSP. In particular, this group reported that PSM-*mec* was expressed in some specific methicillin-resistant isolates of *S. pseudintermedius* and laid the genetic foundation for further elucidating the SCC*mec*-encoded virulence and resistance factors.

For the review/perspective, Uehara [Contribution 2] gave an update on the current status of SCC*mec*. Tenover and Tickler [Contribution 4] commented on the current molecular approaches for rapid detection of MRSA/MSSA in various clinical specimens. De Rose et al. [Contribution 9] reviewed the recent literature on the management of neonatal staphylococcal skin infections and discussed the most appropriate clinical approaches based on four cases of neonatal blistering diseases with staphylococcal infections.

*S. aureus*, including MRSA and MSSA, will remain a major human and animal pathogen. Further research on molecular evolution, epidemiology, characterization, and pathogenicity of staphylococci is needed to obtain a better understanding of the emerging trends in antibiotic resistance and virulence and to therefore control infections caused by this pathogen.

## References

[1] Lakhundi S., Zhang K. (2018). Methicillin-resistant *Staphylococcus aureus*: Molecular characterization, evolution, and epidemiology. Clin. Microbiol. Rev..

[2] Howden B.P., Giulieri S.G., Wong Fok Lung T., Baines S.L., Sharkey L.K., Lee J.Y.H., Hachani A., Monk I.R., Stinear T.P. (2023). *Staphylococcus aureus* host interactions and adaptation. Nat. Rev. Microbiol..

[3] Vestergaard M., Frees D., Ingmer H. (2019). Antibiotic resistance and the MRSA problem. Microbiol. Spectr..

[4] Turner N.A., Sharma-Kuinkel B.K., Maskarinec S.A., Eichenberger E.M., Shah P.P., Carugati M., Holland T.L., Fowler V.G. (2019). Methicillin-resistant *Staphylococcus aureus*: An overview of basic and clinical research. Nat. Rev. Microbiol..

[5] Kirby W.M. (1944). Extraction of a highly potent penicillin inactivator from penicillin resistant staphylococci. Science.

[6] Lowy F.D. (2003). Antimicrobial resistance: The example of *Staphylococcus aureus*. J. Clin. Investig..

[7] Rammelkamp C.H., Maxon T. (1942). Resistance of *Staphylococcus aureus* to the action of penicillin. Proc. Soc. Exp. Biol. Med..

[8] Bondi A., Dietz C.C. (1945). Penicillin resistant staphylococci. Proc. Soc. Exp. Biol. Med..

[9] Jevons M.P. (1961). “Celbenin”-resistant Staphylococci. Br. Med. J..

[10] Thompson R.L., Cabezudo I., Wenzel R.P. (1982). Epidemiology of nosocomial infections caused by methicillin-resistant *Staphylococcus aureus*. Ann. Intern. Med..

[11] Hanifah Y.A., Hiramatsu K., Yokota T. (1992). Characterization of methicillin-resistant *Staphylococcus aureus* associated with nosocomial infections in the University Hospital, Kuala Lumpur. J. Hosp. Infect..

[12] Faoagali J.L., Thong M.L., Grant D. (1992). Ten years’ experience with methicillin-resistant *Staphylococcus aureus* in a large Australian hospital. J. Hosp. Infect..

[13] Cafferkey M.T., Hone R., Coleman D., Pomeroy H., McGrath B., Ruddy R., Keane C.T. (1985). Methicillin-resistant *Staphylococcus aureus* in Dublin 1971-84. Lancet.

[14] Bradley J.M., Noone P., Townsend D.E., Grubb W.B. (1985). Methicillin-resistant *Staphylococcus aureus* in a London hospital. Lancet.

[15] Torvaldsen S., Roberts C., Riley T.V. (1999). The continuing evolution of methicillin-resistant *Staphylococcus aureus* in Western Australia. Infect. Control Hosp. Epidemiol..

[16] Tiemersma E.W., Bronzwaer S.L., Lyytikainen O., Degener J.E., Schrijnemakers P., Bruinsma N., Monen J., Witte W., Grundman H. (2004). European Antimicrobial Resistance Surveillance System Participants. Methicillin-resistant *Staphylococcus aureus* in Europe, 1999–2002. Emerg. Infect. Dis..

[17] Takizawa Y., Taneike I., Nakagawa S., Oishi T., Nitahara Y., Iwakura N., Ozaki K., Takano M., Nakayama T., Yamamoto T. (2005). A Panton-Valentine leucocidin (PVL)-positive community-acquired methicillin-resistant *Staphylococcus aureus* (MRSA) strain, another such strain carrying a multiple-drug resistance plasmid, and other more-typical PVL-negative MRSA strains found in Japan. J. Clin. Microbiol..

[18] Song J.H., Hsueh P.R., Chung D.R., Ko K.S., Kang C.I., Peck K.R., Yeom J.S., Kim S.W., Chang H.H., Kim Y.S. (2011). Spread of methicillin-resistant *Staphylococcus aureus* between the community and the hospitals in Asian countries: An ANSORP study. J. Antimicrob. Chemother..

[19] Rountree P.M., Beard M.A. (1968). Hospital strains of *Staphylococcus aureus*, with particular reference to methicillin-resistant strains. Med. J. Aust..

[20] Kerttula A.M., Lyytikainen O., Karden-Lilja M., Ibrahem S., Salmenlinna S., Virolainen A., Vuopio-Varkila J. (2007). Nationwide trends in molecular epidemiology of methicillin-resistant *Staphylococcus aureus*, Finland, 1997–2004. BMC Infect. Dis..

[21] Kayaba H., Kodama K., Tamura H., Fujiwara Y. (1997). The spread of methicillin-resistant *Staphylococcus aureus* in a rural community: Will it become a common microorganism colonizing among the general population?. Surg. Today.

[22] Griffiths C., Lamagni T.L., Crowcroft N.S., Duckworth G., Rooney C. (2004). Trends in MRSA in England and Wales: Analysis of morbidity and mortality data for 1993–2002. Health Stat. Q..

[23] Givney R., Vickery A., Holliday A., Pegler M., Benn R. (1998). Evolution of an endemic methicillin-resistant *Staphylococcus aureus* population in an Australian hospital from 1967 to 1996. J. Clin. Microbiol..

[24] Enright M.C., Robinson D.A., Randle G., Feil E.J., Grundmann H., Spratt B.G. (2002). The evolutionary history of methicillin-resistant *Staphylococcus aureus* (MRSA). Proc. Natl. Acad. Sci. USA.

[25] Rodríguez-Noriega E., Seas C., Guzmán-Blanco M., Mejía C., Alvarez C., Bavestrello L., Zurita J., Lebarca J., Luna C.M., Salles M.J.C. (2010). Evolution of methicillin-resistant *Staphylococcus aureus* clones in Latin America. Int. J. Infect. Dis..

[26] Moodley A., Oosthuysen W.F., Duse A.G., Marais E. (2010). South African MRSA Surveillance group. Molecular characterization of clinical methicillin-resistant *Staphylococcus aureus* isolates in South Africa. J. Clin. Microbiol..

[27] Ghaznavi-Rad E., Nor Shamsudin M., Sekawi Z., Khoon L.Y., Aziz M.N., Hamat R.A., Othman N., Chong P.P., van Belkum A., Ghasemzadeh-Moghaddam H. (2010). Predominance and emergence of clones of hospital-acquired methicillin-resistant *Staphylococcus aureus* in Malaysia. J. Clin. Microbiol..

[28] Feil E.J., Cooper J.E., Grundmann H., Robinson D.A., Enright M.C., Berendt T., Peacock S.J., Smith J.M., Murphy M., Sratt B.G. (2003). How clonal is *Staphylococcus aureus*?. J. Bacteriol..

[29] D’Souza N., Rodrigues C., Mehta A. (2010). Molecular characterization of methicillin-resistant *Staphylococcus aureus* with emergence of epidemic clones of sequence type (ST) 22 and ST 772 in Mumbai, India. J. Clin. Microbiol..

[30] Chen H., Liu Y., Jiang X., Chen M., Wang H. (2010). Rapid change of methicillin-resistant *Staphylococcus aureus* clones in a Chinese tertiary care hospital over a 15-year period. Antimicrob. Agents Chemother..

[31] Chambers H.F., Deleo F.R. (2009). Waves of resistance: *Staphylococcus aureus* in the antibiotic era. Nat. Rev. Microbiol..

[32] Campanile F., Bongiorno D., Borbone S., Stefani S. (2010). Methicillin-resistant *Staphylococcus aureus* evolution: The multiple facets of an old pathogen. Eur. Infect. Dis..

[33] Breurec S., Fall C., Pouillot R., Boisier P., Brisse S., Diene-Sarr F., Djibo S., Etienne J., Fonkoua M.C., Perrier-Gros-Claude J.D. (2011). Epidemiology of methicillin-susceptible *Staphylococcus aureus* lineages in five major African towns: High prevalence of Panton-Valentine leukocidin genes. Clin. Microbiol. Infect..

[34] Udo E.E., Pearman J.W., Grubb W.B. (1993). Genetic analysis of community isolates of methicillin-resistant *Staphylococcus aureus* in Western Australia. J. Hosp. Infect..

[35] Otter J.A., French G.L. (2011). Community-associated meticillin-resistant *Staphylococcus aureus* strains as a cause of healthcare-associated infection. J. Hosp. Infect..

[36] Elston D.M. (2007). Community-acquired methicillin-resistant *Staphylococcus aureus*. J. Am. Acad. Dermatol..

[37] David M.Z., Glikman D., Crawford S.E., Peng J., King K.J., Hostetler M.A., Boyle-Vavra S., Daum R.S. (2008). What is community-associated methicillin-resistant *Staphylococcus aureus*?. J. Infect. Dis..

[38] David M.Z., Daum R.S. (2010). Community-associated methicillin-resistant *Staphylococcus aureus*: Epidemiology and clinical consequences of an emerging epidemic. Clin. Microbiol. Rev..

[39] Coombs G.W., Pearson J.C., O’Brien F.G., Murray R.J., Grubb W.B., Christiansen K.J. (2006). Methicillin-resistant *Staphylococcus aureus clones*, Western Australia. Emerg. Infect. Dis..

[40] Centers for Disease Contol and Prevention (1999). Four pediatric deaths from community-acquired methicillin-resistant *Staphylococcus aureus*-Minnesota and North Dakota, 1997–1999. MMWR Morb. Mortal. Wkly. Rep..

[41] Weese J.S. (2010). Methicillin-resistant *Staphylococcus aureus* in animals. ILAR J..

[42] Graveland H., Wagenaar J.A., Bergs K., Heesterbeek H., Heederik D. (2011). Persistence of livestock associated MRSA CC398 in humans is dependent on intensity of animal contact. PLoS ONE.

[43] Cuny C., Friedrich A., Kozytska S., Layer F., Nubel U., Ohlsen K., Strommenger B., Walther B., Wieler L., Witte W. (2010). Emergence of methicillin-resistant *Staphylococcus aureus* (MRSA) in different animal species. Int. J. Med. Microbiol..

[44] Wolk D.M., Struelens M.J., Pancholi P., Davis T., Della-Latta P., Fuller D., Picton E., Dickenson R., Denis O., Johnson D. (2009). Rapid detection of *Staphylococcus aureus* and methicillin-resistant *S. aureus* (MRSA) in wound specimens and blood cultures: Multicenter preclinical evaluation of the Cepheid Xpert MRSA/SA skin and soft tissue and blood culture assays. J. Clin. Microbiol..

[45] Whitby M., McLaws M.L., Berry G. (2001). Risk of death from methicillin-resistant *Staphylococcus aureus* bacteraemia: A meta-analysis. Med. J. Aust..

[46] Thampi N., Showler A., Burry L., Bai A.D., Steinberg M., Ricciuto D.R., Bell C.M., Morris A.M. (2015). Multicenter study of health care cost of patients admitted to hospital with *Staphylococcus aureus* bacteremia: Impact of length of stay and intensity of care. Am. J. Infect. Control.

[47] Fortuin-de Smidt M.C., Singh-Moodley A., Badat R., Quan V., Kularatne R., Nana T., Lekalakala R., Govender N.P., Perovic O., for GERMS-SA (2015). *Staphylococcus aureus* bacteraemia in Gauteng academic hospitals, South Africa. Int. J. Infect. Dis..

[48] Antonanzas F., Lozano C., Torres C. (2015). Economic features of antibiotic resistance: The case of methicillin-resistant *Staphylococcus aureus*. Pharmacoeconomics.

[49] Rupp M.E., Archer G.L. (1994). Coagulase-negative staphylococci: Pathogens associated with medical progress. Clin. Infect. Dis..

[50] Banerjee S.N., Emori T.G., Culver D.H., Gaynes R.P., Jarvis W.R., Horan T., Edwards J.R., Tolson J., Henderson T., Martone W.J. (1991). Secular trends in nosocomial primary bloodstream infections in the United States, 1980-1989. National Nosocomial Infections Surveillance System. Am. J. Med..

[51] Otto M. (2013). Coagulase-negative staphylococci as reservoirs of genes facilitating MRSA infection: Staphylococcal commensal species such as *Staphylococcus epidermidis* are being recognized as important sources of genes promoting MRSA colonization and virulence. Bioessays.

[52] Feng Y., Chen C.J., Su L.H., Hu S., Yu J., Chiu C.H. (2008). Evolution and pathogenesis of *Staphylococcus aureus*: Lessons learned from genotyping and comparative genomics. FEMS Microbiol. Rev..

